# Morpho-Physiological Responses of *Arabidopsis thaliana* L. to the LED-Sourced CoeLux^®^ System

**DOI:** 10.3390/plants10071310

**Published:** 2021-06-28

**Authors:** Peter Beatrice, Mattia Terzaghi, Donato Chiatante, Gabriella Stefania Scippa, Antonio Montagnoli

**Affiliations:** 1 Department of Biotechnology and Life Sciences, University of Insubria, 21100 Varese (VA), Italy; donato.chiatante@uninsubria.it (D.C.); antonio.montagnoli@uninsubria.it (A.M.); 2 Department of Chemistry and biology ‘A. Zambelli’, University of Salerno, 84084 Fisciano (SA), Italy; mterzaghi@unisa.it; 3 Department of Biosciences and Territory, University of Molise, 86090 Pesche (IS), Italy; scippa@unimol.it

**Keywords:** CoeLux^®^, LEDs, light intensity, light spectrum, *Arabidopsis thaliana*, photomorphogenesis, growth and development, confined environment, low light

## Abstract

The CoeLux^®^ lighting system reproduces the true effect of natural sunlight entering through an opening in the ceiling, with a realistic sun perceived at an infinite distance surrounded by a clear blue sky. It has already been demonstrated that this new lighting system generates long-term positive effects on human beings; however, there are no investigations so far concerning the plant responses to CoeLux^®^ lighting. To fill this gap, the model plant *Arabidopsis thaliana* L. was grown at four different distances from the light source, corresponding to four different light intensities (120, 70, 30, 20 μmol m^−2^ s^−1^). High-pressure sodium lamps were used as control light. Plant phenology and morpho-physiological traits were monitored to assess for the first time the ability of plants to grow and develop under the light spectrum and intensity of the CoeLux^®^ system. Plants grown at the lower light intensities showed a delayed life cycle and were significantly smaller than plants grown with more light. Furthermore, plants grown under the CoeLux^®^ light type showed an additional deficit when compared to control plants. Overall, our results show that both the light spectrum and intensity of the CoeLux^®^ system had a strong impact on *A. thaliana* growth performance.

## 1. Introduction

Historically, several lighting systems have been used for indoor plant growth, among them fluorescent lamps, metal-halide, high-pressure sodium (HPS), and incandescent lamps [1]. These different light types share common negative features like huge energy consumption, short lifetime, and unwanted heat generation [2]. Recently, the lighting industry has seen rapid growth and the introduction of several new lighting systems. One of the most interesting and quickly developing are light-emitting diodes (LEDs), which show high efficiency, long lifetime, and negligible heat emission [3]. Furthermore, LEDs allow an enormous variety of lighting effects to be produced, among these, the CoeLux^®^ lighting system is one of the last arrivals on the market [4]. CoeLux^®^ system is an innovative LED-based technology for indoor lighting that uses nanostructured materials and optical systems to reproduce Rayleigh scattering effect that occurs when light crosses the earth’s atmosphere [5]. Furthermore, CoeLux^®^ is able to simulate the visual effect of the sun in a blue sky and project realistic shadows in the room. The key difference with other artificial lighting systems is that CoeLux^®^ provides a real impression of natural sunlight together with all its properties [6]. Thus far, the numerous applications of the CoeLux^®^ system include the lighting of hospital wards, subway systems, underground rooms and offices, and, in general, all those spaces that are not naturally illuminated. Furthermore, there is an increasing interest in the possible effects of the CoeLux^®^ lighting systems on human health, in particular on human mood, cognition, and physiological reactions. It has already been demonstrated that this artificial skylight generates positive long-term psycho-physiological effects on human beings comparable to the real counterpart [7].

On the other hand, there are no investigations so far concerning plant responses to CoeLux^®^ lighting. The great suitability of CoeLux^®^ technology for closed or underground environments raises the question of whether this lighting system could be appropriate to grow crop plants for human subsistence [8] or ornamental plants for human well-being [9]. In this context, it must be taken into account that both the quality [10] and quantity [11] of visible light received by plants are crucial for their growth and development. Terrestrial green plants absorb photons unevenly across the electromagnetic spectrum, and only photosynthetically active radiation (PAR) is used to carry out photosynthesis [12]. The photosynthetic pigments in the chloroplasts respond mainly to blue (400–490 nm) and red light (590–700 nm), whereas green and yellow light (490–590 nm) is considerably less efficient in driving photosynthesis [13]. Moreover, in the natural environment, every species of plants is adapted to manage a certain variety of light intensity [14], as in the sunbeam the radiation can easily reach values of 1000–2000 μmol m^−2^s^−1^, whereas in the shade, radiation intensity can lower down to 10–20 μmol m^−2^s^−1^ [15]. Several features of plant form, physiology, and resource allocation vary with the level of irradiance to which plants are acclimated [16]. Plant species adapted to live at a high light intensity show a shade avoidance response when they grow at low light intensity [17].

The interest for further development of the CoeLux^®^ technology continues to grow due to its application in a wide range of artificially illuminated environments. In this context, it is crucial to understand how plants react to this peculiar lighting system and assess if this artificial skylight could sustain plant growth in underground or confined environments. We hypothesized that the low light intensity of these systems could be the principal limit for their use for plant growth, while the light spectrum might affect plant growth only marginally. To test our hypothesis, *Arabidopsis thaliana* plants were grown at four different distances from the CoeLux^®^ system light source, each of them corresponding to different light intensities (120, 70, 30, 20 μmol m^−2^ s^−1^). High-pressure sodium (HPS) lamps, historically considered as an ideal light source for indoor plant growth [18,19], were used to provide a control light type in our study.

## 2. Results

### 2.1. Phenological Analysis

**Figure 1:**
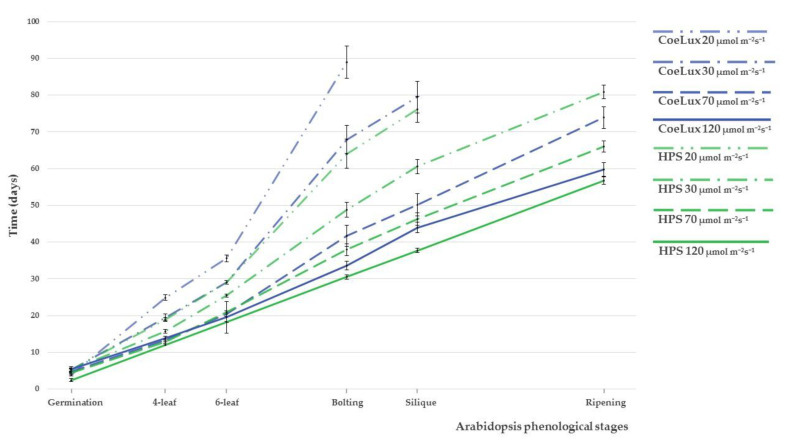
Phenological stages observation were recorded both under CoeLux^®^ light (blue) and under HPS light (green) at four different light intensities, namely 20, 30, 70, and 120 μmol m^−2^s^−1^. Error bars represent the 95% confidence interval.

*Arabidopsis thaliana* plants grown with 120 μmol m^−2^ s^−1^ under the HPS light type (control) completed their life cycle, from sowing to the fruit ripening and senescence phenological stage, in 57 days (dark green solid line in Figure 1). Plants delayed their life cycle completion when growing with lower light intensity (Figure 1). In particular, life cycle duration was inversely related to light intensity (dashed lines). This delay was even wider during the reproductive phase (bolting to ripening stage). Although a similar delay can be observed between control (HPS) and treated plants (CoeLux^®^), the latter plants showed a higher magnitude for all light intensities considered. Significant differences between plants grown under the two different light types increased with the lowering of the light intensity, showing the smaller delay at 120 μmol m^−2^ s^−1^ and the highest delay at 20 μmol m^−2^s^−1^. Furthermore, at the lowest light condition (20 μmol m^−2^ s^−1^), *A. thaliana* plants were unable to complete their life cycle with the production of ripe seeds, both under the HPS light type and the CoeLux^®^ system’s light type. In particular, under the CoeLux^®^ light type, seeds were produced only at the highest light intensities (70 and 120 μmol m^−2^ s^−1^). These seeds were viable and germinated regularly at 98% when sown (data not shown).

### 2.2. Morphological Traits

**Figure 2:**
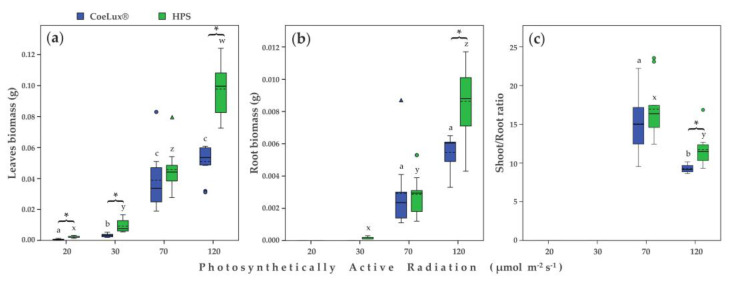
(**a**) Leaves biomass (g), (**b**) root biomass (g), and (**c**) shoot-to-root ratio for different light intensities. Blue and green bars indicate data of plants grown under the CoeLux^®^ and the HPS light type, respectively. Black asterisks indicate statistically significant differences (*p* < 0.05) between plants grown under the CoeLux^®^ and the HPS light type within the same light intensity. Letters indicate statistically significant differences (*p* < 0.05) between plants grown under different light intensities within the same light type. Vertical boxes represent approximately 50% of the observations, and lines extending from each box are the upper and lower 25% of the distribution. Within each box, the solid horizontal line is the median value, whereas the dotted horizontal line is the mean.

The biomass of both leaves and roots was found to increase with the increase of the light intensity (Figure 2a,b). The highest biomass values were measured for plants growing at 120 μmol m^−2^s^−1^, for both leaves (Figure 2a) and roots (Figure 2b) organs. For both leaves and roots biomass, significant differences between plants grown under the CoeLux^®^ light type and plants grown under the HPS light type were measured at 20, 30, and 120 μmol m^−2^s^−1^. The root biomass of plants grown at 20 μmol m^−2^s^−1^ and 30 μmol m^−2^s^−1^ was not measured due to the low weight, which was lower than the limit of the scale range (0.0001 g). The shoot/root ratio data (Figure 2c) were significantly higher in plants grown with 70 μmol m^−2^s^−1^. Moreover, at 120 μmol m^−2^s^−1^, plants grown under the CoeLux^®^ light type showed a significantly lower shoot/root ratio (Figure 2c).

**Figure 3:**
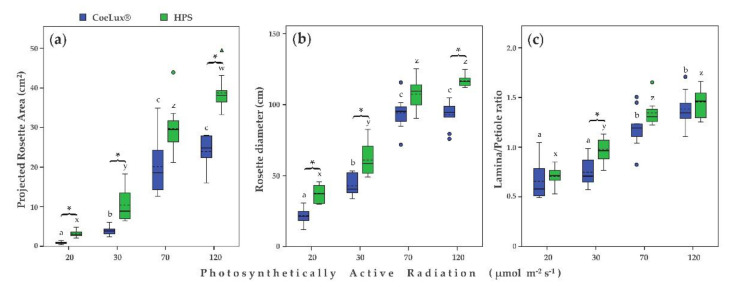
(**a**) Projected rosette area (cm^2^), (**b**) rosette diameter (cm), and (**c**) lamina-to-petiole length ratio for different light intensities. Blue and green bars indicate data of plants grown under the CoeLux^®^ and the HPS light types, respectively. The lamina-to-petiole length ratio is the mean of three leaves for each of the ten replicates. Black asterisks indicate statistically significant differences (*p* < 0.05) between plants grown under the CoeLux^®^ and the HPS light type within the same light intensity. Letters indicate statistically significant differences (*p* < 0.05) between plants grown under different light intensities within the same light type. Vertical boxes represent approximately 50% of the observations and lines extending from each box are the upper and lower 25% of the distribution. Within each box, the solid horizontal line is the median value, whereas the dotted horizontal line is the mean.

The Projected Rosette Area (PRA) increased with the increase in light intensity independently of the light type analyzed (Figure 3a). The only exception was found for the CoeLux^®^ light type, with no differences in PRA between 70 and 120 μmol m^−2^ s^−1^ (Figure 3a). Plants grown under the HPS light type had significantly higher PRA values than plants grown under the CoeLux^®^ light type, with the only exception at 70 μmol m^−2^ s^−1^ (Figure 3a).

The diameter of the rosette (RD) increased with the increase in the light intensity independently of the light type analyzed (Figure 3b). Plants grown under both light types did not show significant differences in RD between 70 and 120 μmol m^−2^ s^−1^ (Figure 3b). Plants grown under the HPS light type had significantly higher values of RD than plants grown under the CoeLux^®^ light type with the only exception at 70 μmol m^−2^ s^−1^ (Figure 3b).

The lamina to petiole ratio (L/P) increased with the increase in light intensity independently of the light type analyzed (Figure 3c). In the case of plants grown under the CoeLux^®^ light type, similar values were measured between 20 and 30 μmol m^−2^s^−1^ and between 70 and 120 μmol m^−2^s^−1^ (Figure 3c). In the case of plants grown under the HPS light type, L/P values were similar between 70 and 120 μmol m^−2^s^−1^. Plants grown under the HPS light type had significantly higher L/P values than plants grown under the CoeLux^®^ light type only at 30 μmol m^−2^s^−1^ (Figure 3c).

### 2.3. Physiological Measurements

**Figure 4:**
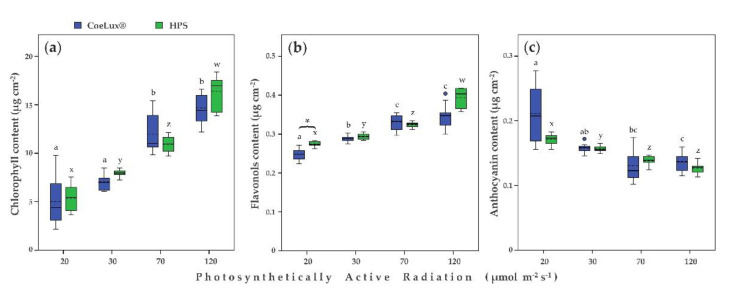
(**a**) Chlorophyll content (µg cm^−2^), (**b**) flavonols content (µg cm^−2^), and (**c**) anthocyanin content (µg cm^−2^) for different light intensities. Blue and green bars indicate data of plants grown under the CoeLux^®^ and the HPS light types, respectively. Black asterisks indicate statistically significant differences (*p* < 0.05) between plants grown under the CoeLux^®^ and the HPS light type within the same light intensity. Letters indicate statistically significant differences (*p* < 0.05) between plants grown under different light intensities within the same light type. Vertical boxes represent approximately 50% of the observations and lines extending from each box are the upper and lower 25% of the distribution. Within each box, the solid horizontal line is the median value, whereas the dotted horizontal line is the mean.

The chlorophyll content increased with the increase in the light intensity, and no significant differences were detected between CoeLux^®^ and control light (Figure 4a). In the case of plants grown under the CoeLux^®^ light type, the highest chlorophyll concentrations were found in plants grown with a light intensity of 70 and 120 μmol m^−2^, while the lowest concentrations were found for 20 and 30 μmol m^−2^ s^−1^, which did not differ from each other (Figure 4a). In the case of plants grown under the HPS light type, the highest and the lowest values were found for 120 and 20 μmol m^−2^ s^−1^, respectively (Figure 4a).

The flavonoid content also increased with the increase in light intensity (Figure 4b). In the case of plants grown under the CoeLux^®^ light type, the highest values were found for 70 and 120 μmol m^−2^ s^−1^ and the lowest values for 20 μmol m^−2^ s^−1^ (Figure 4b). In the case of plants grown under the HPS light type, the highest and the lowest values were found for 120 and 20 μmol m^−2^ s^−1^, respectively (Figure 4b). A significant difference between plants grown under the CoeLux^®^ and the HPS light type was observed only at 20 μmol m^−2^ s^−1^.

The anthocyanin concentration decreased with the increase in light intensity independently of the light type considered (Figure 4c). In the case of plants grown under the CoeLux^®^ light type, the highest and the lowest values were found for 20 and 30 μmol m^−2^ s^−1^ and 70 and 120 μmol m^−2^ s^−1^, respectively. In the case of plants grown under the HPS light type, the highest values were found for 20 μmol m^−2^ s^−1^ and the lowest values for 70 and 120 μmol m^−2^ s^−1^. No significant difference was observed between plants grown under the CoeLux^®^ and the HPS light type, independently of the light intensity considered (Figure 4c).

**Figure 5:**
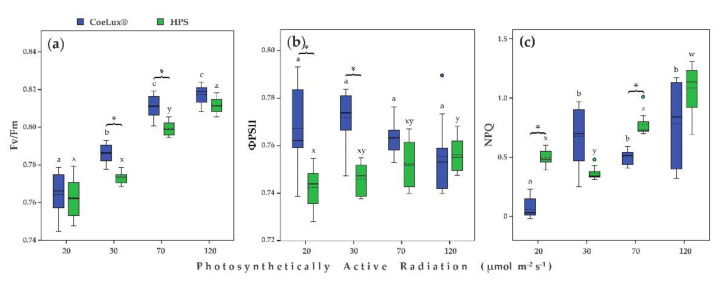
(**a**) Maximum quantum efficiency of PSII photochemistry (*Fv*/*Fm*), (**b**) PSII operating efficiency in the light (ΦPSII), and (**c**) non-photochemical quenching (NPQ) for different light intensities. Blue and green bars indicate data of plants grown under the CoeLux^®^ and the HPS light types, respectively. Black asterisks indicate statistically significant differences (*p* < 0.05) between plants grown under the CoeLux^®^ and the HPS light type within the same light intensity. Letters indicate statistically significant differences (*p* < 0.05) between plants grown under different light intensities within the same light type. Vertical boxes represent approximately 50% of the observations and lines extending from each box are the upper and lower 25% of the distribution. Within each box, the solid horizontal line is the median value, whereas the dotted horizontal line is the mean.

The maximum quantum efficiency of PSII photochemistry (*Fv*/*Fm*) increased with the increase in the light intensity independently of the light type considered (Figure 5a). In the case of plants grown under the CoeLux^®^ light type, *Fv*/*Fm* values were similar at 70 and 120 μmol m^−2^ s^−1^ (Figure 5a). Plants grown under the HPS light type had similar values of *Fv*/*Fm* at 20 and 30 μmol m^−2^ s^−1^. Plants grown under the CoeLux^®^ light type had significantly higher *Fv*/*Fm* values than plants grown under the HPS light type at 30 and 70 μmol m^−2^s^−1^ (Figure 5a).

The PSII operating efficiency in the light (ΦPSII) was not different among different light intensities for plants grown under the CoeLux^®^ light type (Figure 5b). In the case of plants grown under the HPS light type, ΦPSII slightly increased with the increase in the light intensity, with the highest and lowest values measured at 20 and 120 μmol m^−2^s^−1^, respectively (Figure 5b). Plants grown under the CoeLux^®^ light type at 20 and 30 μmol m^−2^s^−1^ had significantly higher ΦPSII values than plants grown under the HPS light type (Figure 5b).

The Non-Photochemical Quenching (NPQ) for plants grown under the CoeLux^®^ light type was not different among different light intensities at 30, 70, and 120 μmol m^−2^s^−1^, while a significantly lower value was observed at 20 μmol m^−2^s^−1^. In the case of plants grown under the HPS light type, NPQ increased with the increase in light intensity, with the only exception of 30 μmol m^−2^s^−1^, which was the lower value, while the highest value was observed at 120 μmol m^−2^s^−1^. Plants grown under the CoeLux^®^ light type at 20 and 70 μmol m^−2^s^−1^ had significantly lower NPQ values than plants grown under HPS light type.

**Figure 6:**
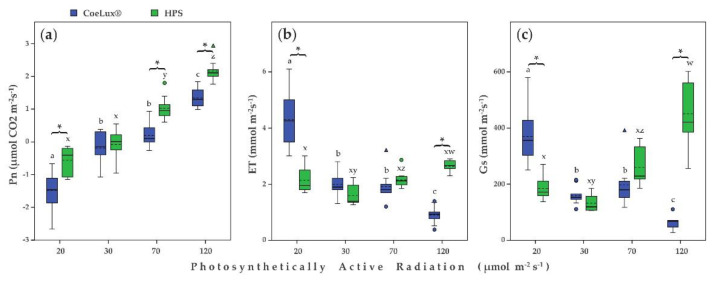
(**a**) Pn: net photosynthetic rate (μmol CO_2_ m^−2^s^−1^), (**b**) ET: evapotranspiration (mmol m^−2^s^−1^), and (**c**) Gs: stomatal conductance (mmol m^−2^s^−1^) for different light intensities. Blue and green bars indicate data of plants grown under the CoeLux^®^ and the HPS light types, respectively. Black asterisks indicate statistically significant differences (*p* < 0.05) between plants grown under the CoeLux^®^ and the HPS light type within the same light intensity. Letters indicate statistically significant differences (*p* < 0.05) between plants grown under different light intensities within the same light type. Vertical boxes represent approximately 50% of the observations and lines extending from each box are the upper and lower 25% of the distribution. Within each box, the solid horizontal line is the median value, whereas the dotted horizontal line is the mean.

The net photosynthetic rate (Pn) increased with the increase of the light intensity independently of the light type considered (Figure 6a). For plants grown under both CoeLux^®^ and HPS light type, the highest and lowest Pn values were measured at 120 and 20 μmol m^−2^s^−1^, respectively (Figure 6a). At 20 and 30 μmol m^−2^s^−1^, negative photosynthetic values were measured due to the glass delimiting the instrument cuvette chamber, which lowered the incident light received by the encapsulated leaf of 52.9 ± 7.3 μmol m^−2^s^−1^. Plants grown under the CoeLux^®^ light type had significantly lower Pn values than plants grown under the HPS light type at 20, 70, and 120 μmol m^−2^s^−1^.

The evapotranspiration rate (ET) for plants grown under the CoeLux^®^ light type decreased with the increase in the light intensity (Figure 6b). The highest and lowest values were found for plants grown, respectively, at 20 and 120 μmol m^−2^s^−1^, while intermediate values were found at 30 and 70 μmol m^−2^s^−1^. In the case of plants grown under the HPS light type, ET values did not differ among different light intensities (Figure 6b). The ET values measured at 20 μmol m^−2^s^−1^ for plants grown under the CoeLux^®^ light type were significantly higher than the values measured for plants grown under the HPS light type (Figure 6b). The ET values measured at 120 μmol m^−2^s^−1^ were significantly higher for plants grown under the HPS light type than plants grown under the CoeLux^®^ light type (Figure 6b).

The stomatal conductance (Gs) decreased with the increase in the light intensity in the case of plants grown under the CoeLux^®^ light type (Figure 6c). The highest and lowest Gs values were measured, respectively, at 20 and 120 μmol m^−2^s^−1^, while intermediate values were found at 30 and 70 μmol m^−2^s^−1^. In the case of plants grown under the HPS light type, the Gs values were not different among light intensities, with the only exception of 120 μmol m^−2^s^−1^, which showed the highest values (Figure 6c). At 20 μmol m^−2^s^−1^, the Gs value measured for plants grown under the CoeLux^®^ light type was significantly higher than the value measured for plants grown under the HPS light type (Figure 6c). On the contrary, at 120 μmol m^−2^s^−1^, the Gs value measured for plants grown under the HPS light type was significantly higher than the value measured for plants grown under the CoeLux^®^ light type (Figure 6c).

## 3. Discussion

In our study, we used the model plant *Arabidopsis thaliana* to assess for the first time if the light spectrum and intensity of the CoeLux^®^ 45HC lighting system could be suitable for plant growth in controlled environments. Both light quantity and quality are fundamental for plant growth and development [20]. In this context, LEDs show unrivaled advantages, since LED bulbs can be assembled in countless ways to obtain exactly the light characteristics needed for optimal plant growth [10]. However, the CoeLux systems have peculiar constraints due to the physical effects involved in the setting up of their characteristic visual effects [4,6]. Thus, light quantity and quality cannot be adjusted like with other LED-based lighting systems currently used for plant growth [10]. We observed that the light emitted by the 45HC CoeLux^®^ system, even inside the sunbeam, was characterized by low levels of photosynthetic active radiation (PAR). The registered values were similar to those that can be normally found in shaded environments, for example, under a dense forest canopy [21]. Consequently, even if natural sunlight’s visual effects were perfectly reproduced, this artificial skylight cannot be compared to its natural counterpart in terms of light intensity. Shade-adapted plants are certainly the most suitable to grow under this lighting system, as photosynthesis is directly influenced by the amount of light reaching the plant’s leaves [16].

With the phenological analysis, we observed that *A. thaliana* plants grown under the CoeLux^®^ light type showed a significant delay with respect to plants grown under the HPS light type, and this delay was independent of the light intensities considered. Moreover, this plant development delay was particularly evident at the last growth stages such as *Bolting* and *Silique* set, and it was of higher magnitude at the lowest light intensities. In particular, plants grown at 20 and 30 µmol m^−2^s^−1^ could not reach the seed maturity stage during the 100-days period analyzed in our study. Other studies also reported a 2-week flowering delay in *A. thaliana* plants grown under reduced light intensity and lowered R/FR [22]. Morphological data are in line with the phenological observations, highlighting the negative influence of the CoeLux^®^ light type on *A. thaliana* growth. In fact, for all morphological parameters analyzed, we observed a similar trend that grows with the increase in the light intensity but was always slightly lower with the CoeLux^®^ light type than with the HPS light type.

Plants have to balance the biomass allocation to leaves, stems, and roots in a way that matches the physiological functions performed by these organs. In stress situations, plants allocate relatively more biomass to roots if the limiting factor for growth is below ground (e.g., nutrients or water), whereas they will allocate relatively more biomass to shoots if the limiting factor is above ground (e.g., light or CO_2_) [23]. That is, plants that received a lower irradiance showed increased allocation to the shoots in an attempt to enhance the uptake to the most limiting factor, light. Surprisingly, plants grown under CoeLux^®^ light type showed slightly lower shoot–root ratios relative to control plants.

In addition to biomass, also the PRA and the RD showed a clear detrimental effect of the CoeLux^®^ light type with respect to the HPS light type, demonstrating that this light type is less appropriate than the control one for *A. thaliana* plants growth. This effect could be explained by the different fractions of blue and red light radiated by the two light types, as the blue and red components represent 59% of the total irradiation under the HPS light type and only 55% under the CoeLux^®^ light type (Figure 9). The CoeLux^®^ light type showed a higher yellow component (+4%); however, yellow light is less efficient in driving photosynthesis, as plant’s photosystems respond mainly to red and blue light.

In *A. thaliana*, the lamina to petiole ratio is one of the principal indicators of shade avoidance syndrome (SAS) [24]. In low light conditions, plants grew a longer petiole and a shorter lamina in an effort to collect more light, consequently decreasing the L/P ratio below 1.0. Furthermore, plants that were grown under the CoeLux^®^ light type showed slightly lower L/P ratios than control plants, indicating the onset of a more severe shade avoidance syndrome (SAS) caused by the light quality. Specifically, the CoeLux^®^ light type is characterized by a lower blue component and a lower B/G ratio (Figure 9), which could trigger an SAS via the cryptochrome pathway [24,25]. In natural environments, light reflected or transmitted through photosynthetic tissues of plants in close proximity is depleted in blue, red, and UV-B wavelengths. Therefore, the reflected or transmitted light is enriched in green and far-red spectral regions, resulting in lowered R/FR and B/G ratios. Plants perceive these differences through multiple photoreceptors to regulate shade avoidance responses and tune the plant growth under suboptimal light environments [17].

During shade avoidance responses, many aspects of leaf development are modified, including pigment production [26]. The chlorophyll content is known to decrease at low light intensities [27,28]. A pattern of this nature was also observed in our experiment, with no significant differences between the two different light types analyzed. Flavonoids, such as flavonols and anthocyanins, are also involved in plant’s responses to light stress, as they were proposed to protect against high irradiance, both UV and visible [29]. Furthermore, flavonoids are also antioxidants that can scavenge reactive oxygen species (ROS) and can be observed frequently when plants are exposed to other physiological stresses such as extreme temperatures, drought, or nutritional stresses, in addition to high light and UV radiation [30]. Thus, the biosynthesis of these compounds is regulated by the interplay of multiple factors. Furthermore, the pigment content varies in leaves of different ages [31], and young leaves of many plants have transiently high concentrations of anthocyanins, disappearing as leaves mature [32]. *A. thaliana* plants growing at lower light intensities displayed a strong growth delay (Figure 1); consequently, the pigment concentration measurements were taken on younger leaves with the lowering of light intensity, explaining the unexpected reduction in anthocyanins content observed with the increase in light intensity in *A. thaliana* leaves.

The *Fv*/*Fm* ratio gives a robust indicator of the maximum quantum yield of PSII chemistry and is commonly used to detect plant stress in leaves [33]. Plants grown at lower light intensities showed lower *Fv*/*Fm*, suggesting a stress condition related to light quantity. However, the CoeLux^®^ light type appears to have a positive effect on PSII photochemistry, as we found slightly higher *Fv*/*Fm* values compared to control plants. This observation is probably related to the higher photoinhibition of control plants grown under the HPS light type (Figure 5c), as Murchie et al. reported lowered values of *Fv*/*Fm* in leaves in a quenched state [34]. Nonetheless, an equal reduction in *Fv*/*Fm* was not observed in response to the increased NPQ with the increase in light intensity, suggesting the involvement of multiple factors. The use of leaf samples with different pigment contents may also be a source of inaccuracies [33]. The quantum yield of PSII (Φ_PSII_) showed only minimal differences between the different light intensities, both with the CoeLux^®^ and the HPS light type (Figure 5b).

The drop in light intensity resulted in a lowered net photosynthetic rate in *A. thaliana* plants. Furthermore, the CoeLux^®^ light type negatively influenced the Pn at three of the four light intensities tested, explaining the patterns observed in Figure 2a,b. The lower CO_2_ assimilation under the CoeLux^®^ light type causes a lack of essential building blocks and, consequently, an impaired biomass production. Evapotranspiration rate and stomatal conductance showed similar patterns but no clear differences between the two light types were detected (Figure 5b,c).

Overall, our results showed that the intensity of the light, both under control and CoeLux^®^ light types, had a strong impact on plant growth performance, demonstrating that the light intensity could be the major limiting factor for plants growing under this led-sourced artificial skylight. Furthermore, the light quality of the CoeLux^®^ system showed a negative impact on *A. thaliana* growth, independently of the light intensity considered, demonstrating that light quality could be an additional limiting factor for plants growing under this light source. Further research is needed to assess if shade-tolerant plant species could perform better than *A. thaliana* under this peculiar lighting system, while the comprehension of the molecular mechanisms underlying the observed phenomena could provide significant starting points for the development of CoeLux-adapted plant strains.

## 4. Materials and Methods

### 4.1. Light Characterization

**Figure 7:**
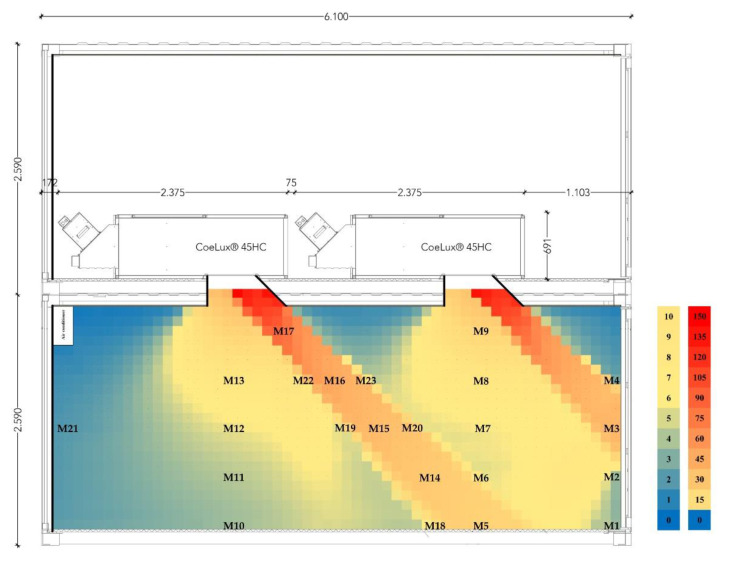
The photosynthetically active radiation (PAR) color-scale map of the double container showroom at Insubria University Campus (Varese, IT). The PAR values in the figure are given in μmol m^−2^s^−1^. Spectra measurements (M) were taken at positions M1 to M23.

The CoeLux^®^ growth room (University of Insubria) is composed of two standard 1 TEU containers assembled one above the other. The upper container hosts the two CoeLux^®^ 45HC lighting systems, while the lower one is insulated and equipped with an air conditioner for temperature control to function as a growth room. The lighting system is sourced by full-spectrum white LEDs with a color temperature of 6500 K. This light is subsequently filtered to obtain the desired skylight effect [6], modifying both spectra and intensity of the original light. Therefore, both light quality and intensity were characterized within a representative section of the growth room (Figure 7).

The HD 2302.0 Light Meter (Delta Ohm) was used to measure the photosynthetically active radiation (PAR) along the central section of the growth room (566 cm × 256 cm). A custom-made rail was designed to guide the instrument sensor along the selected section and perform measurements exactly every 10 cm across the whole surface. The resulting data were analyzed to obtain a color-scale map (Figure 7). The light radiated by the CoeLux^®^ systems is not uniformly diffused inside the growth room, being concentrated within the sunbeam ray of light with a fixed angle of 45°. Within the sunbeam, the highest PAR intensity, measured at 10 cm from the lighting system, was 140 μmol m^−2^s^−1^, while at a further distance, it drops rapidly to around 20 μmol m^−2^s^−1^. These values are even of a lower magnitude when measured within the shade, ranging from 26 μmol m^−2^s^−1^ under the blue sky from the system to less than 1 μmol m^−2^s^−1^ in the most shaded parts of the growth room. Increased light intensity was observed in some shade areas due to light reflection on the walls of the growth room and the frames of the CoeLux^®^ systems skylight (Figure 7).

Spectra measurements every 4 nm in the range between 380 nm and 780 nm were taken on a horizontal white reflector using the SpectraScan PR655 (Photo Research), both inside the CoeLux^®^ growth room and under the HPS lamps that we used as control. Inside the CoeLux^®^ growth room, a total of 23 measurements were performed: 17 of them along the central section of the growth-room at five different heights from the ground floor (0, 50, 100, 150, 200 cm), inside the sunbeam of the CoeLux^®^ system, outside the sunbeam but under the blue panel of the lighting system (sky), and in the deep shade part of the container (Figure 7). The other 6 measurements were taken near the lateral and bottom walls of the growth room to investigate the influence on the light spectra of light reflecting on the grey walls of the growth room. Within the same measurement, the instrument also provides a light intensity value in the form of luminance (cd/m^2^), which was used to normalize the spectra measurements. The spectra were divided into color components: blue light is the integral between 400 and 490 nm, green light is the integral between 490 and 560 nm, yellow light is the integral between 560 and 590 nm, red light is the integral between 590 and 700 nm, and far-red light is the integral between 700 and 780 nm. The red-to-far-red ratio (R/FR) and the blue-to-green ratio (B/R) were calculated according to Sellaro et al. [25].

**Figure 8:**
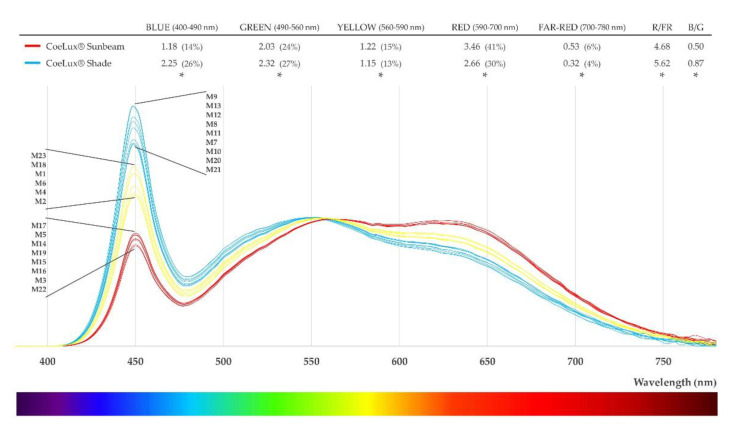
Light spectra measured within the sunbeam (red lines), in shade areas (light blue lines), and in frontier positions between sun and shade (yellow lines). Measurements (M) are reported in order of appearance in the graph. Color components mean values are reported and statistically significant differences are marked with an asterisk (*). R/FR: red/far-red ratio; B/G: blue/green ratio.

We observed only small differences between the light spectrum measured within the sunbeam (red lines in Figure 8) and that measured within the shade (light blue lines in Figure 8), independently of the distance from the light source or room walls. In both cases, the spectra covered almost the entire visible wavebands; however, the total irradiance was differently distributed. Within the sunbeam, the spectra presented a broad peak between 490 and 700 nm and a sharp peak of irradiance of comparable height in the blue region (400–490 nm), representing 14% of the entire irradiance. Within the shade, the spectrum had a similar pattern but with a higher peak at 450 nm (representing 26% of the entire irradiance) and lower values in the red component of light between 590 and 700 nm (30% vs. 41%). Thus, at an equal light intensity, plants placed in a shade position received more blue and green light while plants placed inside the sunbeam receive more red and far-red light. In the small frontier positions between sun and shade, we found spectra with an intermediate shape (yellow lines in Figure 8).

**Figure 9:**
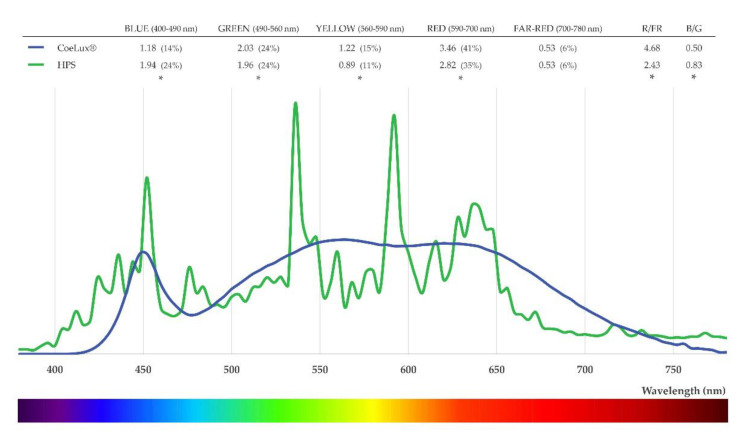
Mean light spectra curves collected within the CoeLux^®^ sunbeam (blue) and under HPS light (green). Color components mean values are reported and statistically significant differences are marked with an asterisk (*). R/FR: red-to-far-red ratio; B/G: blue-to-green ratio.

High-pressure sodium (HPS) lamps (Philips MasterColour CDM-T MW eco 230W/842) were used to provide a control light type in our study. To characterize this light spectrum, a total of 12 spectra measurements were performed at different positions in the range between 120 and 20 μmol m^−2^s^−1^. Data were normalized on luminance to compare the spectra generated by the CoeLux^®^ systems within the sunbeam with those of HPS lamps (Figure 9). The HPS light type were shown to have a higher blue component (24% vs. 14%), while the CoeLux^®^ light type had more yellow (15% vs. 11%) and red (41% vs. 35%) components. The green light component was almost identical even if a statistically significant difference was detected, while the far-red component showed no significant difference between the two, as it represented 6% of the total radiation for both types of light (Figure 9).

### 4.2. Plant Material and Growth Conditions

*Arabidopsis thaliana* wild-type (WT) seeds were stratified at 4 °C for 5 days on 1% agar gel and subsequently transferred to pot flats (Araflats; Arasystem; Ghent/Belgium) composed of 51 individual pot cavities with a 5 cm diameter, filled with sterilized commercial soil-less substrate. Plants were grown at a temperature as close as possible to 22 °C, with an air humidity ranging between 50% and 70%, and a photoperiod of 14 h. A constant 1-cm water layer was maintained in the tray and 1mL liquid fertilizer (NPK 7.5–3−6) was supplied weekly. In the CoeLux^®^ growth room, full pot flats were located at four different positions at progressive distances from the light source (20, 85, 205, 365 cm) inside the system’s sunbeam, each corresponding to a different value of light intensity, respectively 120, 70, 30, 20 μmol m^−2^s^−1^. In our CoeLux^®^ facility, 120 μmol m^−2^s^−1^ is the position suitable for plant growth with the highest light intensity achievable. In a separate growth room, with the same environmental parameters of the CoeLux^®^ growth room, plants were illuminated with HPS lamps as reference light (control), recreating the same light intensity of each of the four positions under the CoeLux^®^ light.

### 4.3. Plant Analysis

Phenological analysis [35] was performed through the recording of different developmental stages for a period of 100 days after sowing, considered as day 0 (Table 1). A total of 10 plants were monitored for each growth condition.

For morphological measurements, a total of 10 *A. thaliana* plants for each growth condition were sampled after 33 days from sowing. The leaves (complete rosette) of the plants were scanned at 800 dpi with the Epson Expression 12000XL instrument and then oven-dried at 70 °C until constant weight. Plant roots were freed from soil media by carefully washing them under running water and subsequently oven-dried until constant weight. The dry organs were then weighed on an analytical balance (Orma AL220S) and the shoot-to-root ratio (S/R) was calculated. The scanned images were processed with WinRhizo (Regent Instrument) to measure the projected rosette area (PRA) and with ImageJ (NIH, USA) to measure the rosette diameter (RD) as well as lamina and petiole length of three leaves for each plant. The lamina-to-petiole ratio (L/P) was then calculated.

Physiological measurements were conducted as follows on 10 different plants for each growth condition.

(A) Chlorophyll fluorescence analysis was performed with a modulated chlorophyll fluorometer (OS1-FL; Opti-Sciences) after 35 days from sowing. The maximum quantum efficiency of photosystem II (PSII) photochemistry (*Fv*/*Fm*) was measured pre-dawn, while the PSII operating efficiency (ΦPSII) was measured after at least 3 h of plant exposure to light. Non-photochemical quenching (NPQ) was subsequently calculated using Fm and Fm’ values [33,36].

(B) The leaf pigment content was measured on the upper face of completely expanded young leaves with the Dualex Scientific Instrument (Force-A) 36 days after sowing. The concentrations of chlorophyll, anthocyanins, and flavonoids are reported by the instrument in µg/cm^2^.

(C) The Ciras 2 instrument (PP Systems) was used to measure the net photosynthetic rate (Pn), the stomatal conductance (Gs), and the evapotranspiration rate (ET) 48 days after sowing. The single leaf gas exchange measurements were taken under ambient light with the 25 × 7 mm leaf cuvette oriented perpendicularly to the light source. At least three measurements for each leaf were taken on completely expanded young leaves. For leaves smaller than 25 × 7 mm, digital pictures were made to determine the projected leaf area inside the cuvette and properly scale the measurement [37].

### 4.4. Statistical Analysis

Statistical analysis was carried out with SPSS 20.0 (SPSS Inc). The post hoc Dunnett’s test was used for multiple comparisons, while the Student’s t-test was used when only two means were compared. Statistically significant differences (*p* < 0.05) between the means were marked with the letters a, b, c, d for the CoeLux^®^ light type, with the letters x, y, z, w for the HPS light type, and with a black asterisk for comparisons between the two types of light. In boxplots, colored circles and triangles represent respectively outliers (outside the 3rdQ + 1.5 × IQR and the 1stQ − 1.5 × IQR) and extreme outliers (outside the 3rdQ + 3 × IQR and the 1stQ – 3 × IQR). Microsoft Excel functions were used to show the 95% confidence interval error bars in Figure 1.

## Figures and Tables

**Table 1:** *A. thaliana* growth stages recorded in this work.

Stage	Description
Germination	Plants with fully expanded cotyledons
4-leaf stage	Plants with the first two rosette leaves bigger than the cotyledons
6-leaf stage	Plants with the second couple of rosette leaves bigger than the first one
Bolting and flowering	Plants with a floral stalk taller than 1 cm
Silique formation	Plants with at least one fully developed silique
Ripening and senescence	Plants with at least one silique totally brown or open with ripe seeds

## Data Availability

The data presented in this study are available on request from the corresponding author.
